# A Nationwide Population-Based Study on the Incidence of Parapharyngeal and Retropharyngeal Abscess—A 10-Year Study

**DOI:** 10.3390/ijerph18031049

**Published:** 2021-01-25

**Authors:** Tzong-Hann Yang, Sudha Xirasagar, Yen-Fu Cheng, Chuan-Song Wu, Yi-Wei Kao, Herng-Ching Lin

**Affiliations:** 1 Department of Otorhinolaryngology, Taipei City Hospital, Taipei 10341, Taiwan; tzonghannyang@gmail.com (T.-H.Y.); seancswu@seed.net.tw (C.-S.W.); 2 Department of Speech, Language and Audiology, National Taipei University of Nursing and Health, Taipei 11219, Taiwan; entist@gmail.com; 3 Research Center of Sleep Medicine, College of Medicine, Taipei Medical University, Taipei 11031, Taiwan; 4 General Education Center, University of Taipei, Taipei 10617, Taiwan; 5 Department of Health Services Policy and Management, Arnold School of Public Health, University of South Carolina, Columbia, SC 29208, USA; sxirasagar@sc.edu; 6 Department of Medical Research, Taipei Veterans General Hospital, Taipei 11217, Taiwan; 7 Department of Otolaryngology—Head and Neck Surgery, Taipei Veterans General Hospital, Taipei 11217, Taiwan; 8 Faculty of Medicine, National Yang-Ming University, Taipei 11217, Taiwan; 9 Institute of Brain Science, National Yang-Ming University, Taipei 11217, Taiwan; 10 Big Data Research Center, Taipei Medical University, Taipei 11031, Taiwan; kyw498762030@gmail.com; 11 Sleep Research Center, Taipei Medical University Hospital, Taipei 11031, Taiwan; 12 School of Health Care Administration, College of Management, Taipei Medical University, Taipei 11031, Taiwan

**Keywords:** parapharyngeal abscess, retropharyngeal abscess, incidence, epidemiology

## Abstract

This study aimed to investigate the annual incidence of parapharyngeal and retropharyngeal abscess (PRPA) based on 10-year population-based data. Patients with PRPA were identified from the Taiwan Health Insurance Research Database, a database of all medical claims of a randomly selected, population-representative sample of over two million enrollees of the National Health Insurance system that covers over 99% of Taiwan’s citizens. During 2007–2016, 5779 patients received a diagnosis of PRPA. We calculated the population-wide incidence rates of PRPA by sex and age group (20–44, 45–64, and >64) as well as in-hospital mortality. The annual incidence rate of PRPA was 2.64 per 100,000 people. The gender-specific incidence rates per 100,000 people were 3.34 for males and 1.94 for females with a male:female gender ratio of 1.72. A slight increase in incidence rates among both genders over the study period was noted. Age-specific rates were lowest in the 20–44 age group with a mean annual incidence of 2.00 per 100,000 people, and the highest rates were noted in the age groups of 45–64 and >64 years with mean annual incidences of 3.21 and 3.20, respectively. We found that PRPA is common in Taiwan, males and older individuals are more susceptible to it, and incidence has increased in recent years.

## 1. Introduction

Parapharyngeal and retropharyngeal abscesses (PRPAs) arise at one of two adjacent anatomical spaces in the neck, often collectively termed as deep neck space abscesses [1]. These infections often develop as sequelae of upper respiratory infections and are potentially life-threatening because of the possibility of the bacterial invasion of the carotid sheath and critical structures within it (e.g., common carotid artery, internal jugular vein, and vagus nerve), potential for airway obstruction, and systemic sepsis [2]. The infection may easily spread to other contiguous spaces, especially to the “danger” zones, possibly leading to mediastinitis and death [3]. Patients may suffer from sore throat, trismus, dysphagia, odynophagia, stridor, dyspnea, hoarseness, and unilateral paresis of the tongue. Hospitalization for appropriate intravenous antibiotics and surgical drainage are imperative for a positive outcome [4].

Despite the aforementioned impacts of PRPA and the high cost burden [5], there is a paucity of long-term population-based epidemiological data on the incidence and mortality of PRPA, especially among the Chinese population. Previous studies have documented incidence rates of PRPA, as well as age and gender distributions, longitudinal trends, and in-hospital mortality [2,5,6]. However, some of these studies have not been representative of the population due to small sample sizes and hospital-based study samples [2,6,7,8,9]. Therefore, this study aimed to estimate the incidence of PRPA among the Taiwan population including age and gender distributions, as well as longitudinal trends, using insurance claims data in Taiwan’s National Health Insurance (NHI) databases.

## 2. Materials and Methods

### 2.1. Database

We obtained data from Taiwan’s National Health Insurance Dataset (NHIRD)—published by the National Health Research Institute in Taiwan—covering a period of ten years (from January 2007 to December 2016). The NHIRD consists of curated administrative claims data from the Taiwan NHI program. The NHIRD includes claims data and registration files of over 99% of all Taiwanese residents (*n* = 23.72 million in 2015–2016). The NHIRD provides an excellent opportunity for Taiwan researchers to perform large-scale clinical epidemiology investigations on diseases.

This study was approved by the Institutional Review Board of Taipei Medical University (TMU-JIRB N202011013). This study adhered to the Strengthening the Reporting of Observational Studies in Epidemiology (STROBE) guidelines of research reporting standards [10].

### 2.2. Study Sample

Because the hospitalization of all patients with PRPA is standard medical care policy in Taiwan, we used the NHIRD inpatient database to identify all patients aged ≥20 years with a principal diagnosis of PRPA (International Classification of Diseases, Ninth Revision, Clinical Modification (ICD-9-CM) codes 478.21, 478.22, or 478.24 or ICD-10-CM codes J39.0 or J39.1) admitted between 1 January 2007 and 31 December 2016 to investigate yearly incidence rates and trends of PRPA. We identified a total of 5779 PRPA patients and categorized them into three 3 age groups: 20–44, 45–64, and >64.

### 2.3. Population Data

We obtained population census data from the Population Affairs Administration at Ministry of the Interior in Taiwan, which are publicly released annually, including age and gender distributions. We used the population data to calculate the national incidence rates per 100,000 people and by age and gender over the ten-year study period from January 2007 to December 2016.

### 2.4. Statistical Analysis

The annual PRPA incidence rate was calculated as the sum of new PRPA cases divided by the size of the total Taiwanese population in a year. Furthermore, yearly PRPA incidence rates per 100,000 people were calculated for 10 years, by gender, and for the age groups of 20–44, 45–64, and >64 years. We used a *t*-test to examine the difference in annual PRPA incidence rate between genders. In addition, we used the annual percent change (APC) and 95% confidence intervals (CIs) to quantify trends in annual PRPA incidence using a linear model to determine whether the APC was statistically significantly different from zero.

## 3. Results

A total of 5779 hospital admissions with PRPA occurred between 2007 and 2016, with a mean patient age of 52.16 years (standard deviation = 16.61) and 64.2% male. There were 33.7%, 42.2%, and 24.1% of PRPA patients in the age groups of 20–44, 45–64, and >64 years, respectively. The majority of PRPA patients (48.8%) resided in Northern Taiwan, and only 2.1% were in Southern Taiwan. In-hospital mortality among the study patients over the 10-year period was 2%. Table 1 presents the demographic characteristics of the study patients.

The mean annual incidence rate of PRPA over the 10-year period was 2.64 per 100,000, varying between 2.43 and 2.81 in the first five years and between 2.47 and 3.71 in the later five-year periods (data not shown in tables). However, there was no statistically significant increase in annual incidence rate of PRPA over the study period with an APC of 1.26% (95% CI = −13.28–18.25%).

**Figure 1:**
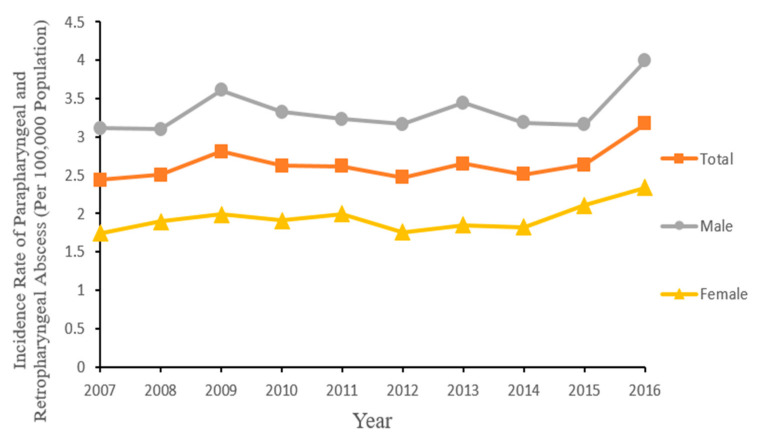
Annual incidence rates of parapharyngeal and retropharyngeal abscess in Taiwan by gender during 2007–2016.

Gender-wise annual incidence rates are shown in Figure 1. The *t*-test revealed that incidence rates among males were consistently and statistically significantly higher compared to females (*p* < 0.001). Gender-specific rates over the study period were 3.34 per 100,000 for males and 1.94 per 100,000 for females, with a male to female gender ratio of 1.72. There was a slight increase in PRPA rates among both genders over the study period.

**Figure 2:**
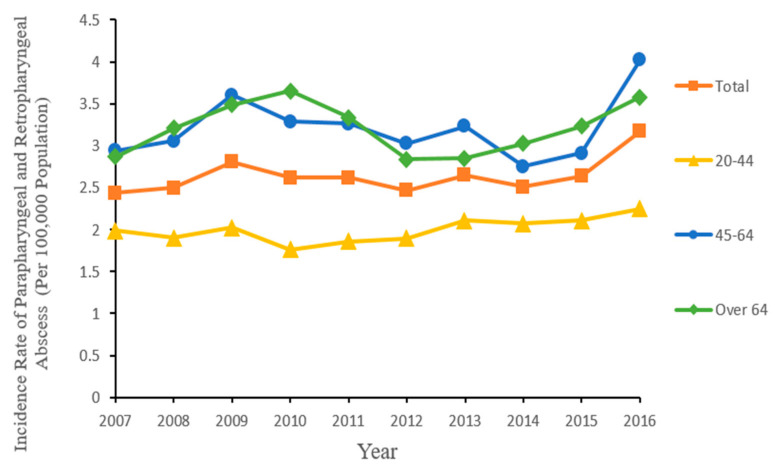
Annual incidence rates of parapharyngeal and retropharyngeal abscess in Taiwan by age group during 2007–2016.

The age-specific incidence over the study period is presented in Table 1. As expected, the age-specific rate was lowest in the 20–44 age group with an average annual incidence of 2.00 per 100,000 over the study period. Incidence rates among the 45–64 and >64 years age groups were similar (3.21 and 3.20, respectively). Figure 2 presents year-wise incidence rates among each age group, showing consistently higher rates among the older age groups compared to distinctly lower rates among younger adults.

## 4. Discussion

Reliable population-based incidence data on PRPA including long-term trends and age and gender distributions have not been published because most available data are from hospital-based case series and therefore not representative. To our knowledge, this was the first population-based survey to investigate the incidence of PRPA using Taiwan’s NHIRD, which includes data on the entire population of Taiwan.

Our study showed a higher mean age of occurrence of PRPA (52.16 years old) than the published literature, which has shown mean ages of 44–48.5 years [5,6,7,11,12]. Our finding of a consistent male preponderance in every study year was in line with previous studies [2,7,11]. We also found incidence differences between age groups that were observed consistently each year over the 10-year study period (Figure 2). Our estimate of an in-hospital mortality rate (around 2.0%) was in line with previous studies that examined mortality rates (reporting rates between 0.3–3.4% of all deep neck infection) [5,6,9,13,14,15,16,17,18]. However, one study from Germany reported no mortality of PRPA in their cohort [19].

Our study showed an annual incidence rate of PRPA of 2.43–3.17 per 100,000 adult people during 2007–2016, which was higher than similar population-based studies. A study from Germany showed a much lower PRPA incidence, 1.32–1.94 per 100,000 people with an incidence of 1–2 per 100,000 above 15 years old during a comparable study period [19]. A Scottish study using the United Kingdom’s National Health Service (NHS) data of 2012 and 2016 that excluded patients aged under 16 years reported annual rates ranging between 0.55 and 2.45 per 100,000 in 2012–2016 for deep neck infections after including infections in the submandibular space (10.8%) and multiple spaces (12.6%) [5]. Another NHS study based on data from England for 1991–2011 estimated PRPA rates of 3–5 per 100,000 with the inclusion of pediatric patients [20]. The discrepancies in incidence rates between our study and other studies may be due to the included age groups, geographic regions, ethnicities, and/or life styles. Notably, both the British NHS and Germany’s health system have a universal health care coverage system with full access to high-quality medical care to all citizens without financial barriers, similar to Taiwan. The higher incidence rate in this study compared with other countries addresses the importance of the disease entity, especially for health care workers’ early alertness. Furthermore, it might warrant further studies of the impact of health system in Taiwan.

Interestingly, during the study period, we found an increasing incidence of PRPA, from 2.43 to 3.17 per 100,000 during our study period, with the increasing trend observed in all age and gender groups. Many recent studies have shown a similar trend [5,11,13,20]. Similarly, the study from Germany reported a 47% increase in the incidence rate of PRPA from 1.32 cases per 100,000 in 2005 to 1.94 per 100,000 in 2017 [12]. The Scottish study also found a similar rising trend for deep neck space infection. A US study demonstrated an increasing incidence rate from 2003 to 2010 with a peritonsillar abscess complicated by a retropharyngeal abscess [11]. Some authors have proposed that older age may be a risk factor for PRPA [6,11]; an aging population may result in increasing trends of PRPA among adults, which may account for our finding. Another potential factor is the trend towards increased tonsillectomy or uvulopalatopharyngoplasty rates in Taiwan [21,22]. Further studies are needed to explore the reasons for the observed increasing trend in PRPA incidence rates.

Infections in retropharyngeal space are easily spread down to the mediastinum and then to the lung and heart, compressing the airway and invading both the carotid artery and jugular vein due to proximity. Widespread infections may easily cause septic shock and disseminated intravascular coagulopathy [23]. Therefore, retropharyngeal space infections are likely to develop life-threatening complications, making PPRP more lethal than deep neck infections [12]. Using a population-based design, our study was ultimately able to calculate an in-hospital mortality for PRPA of 2.0%. The discrepant mortality rates from studies of deep neck infections may be results of unavoidable selection bias brought by hospital-based designs.

Several strengths in our study should be noted. The NHIRD provides an excellent opportunity for Taiwan researchers to perform large-scale clinical epidemiology investigations on diseases. Taiwan’s NHI offers a fully accessible and affordable health care system for every citizen, which limits selection bias in PRPA ascertainment due to socioeconomic status. Furthermore, the NHIRD consists of all claims of the entire Taiwanese population, about 23 million, including outpatient visits, emergency department visits, and inpatient admissions during 2007–2016. Our study identified the PRPA patients by ICD codes from inpatient admission claims. PRPA is a serious and emergent condition that requires patients to seek immediate medical help and hospitalization. The NHI, providing affordable and accessible health care across the nation, enabled us to gather almost all cases of PRPA. As a result, we believe that we included all PRPA patients, regardless of their socioeconomic status. The use of claims data also avoided recall bias and misclassification bias that may result from studies based on self-reported data. However, there have only been three prior population-based studies which used ICD code as a diagnosis for PRPA [11,13,20]. Other such studies have all been hospital-based with findings that may not be generalized to the whole population.

The study had some limitations. Claims data do not have medical data on disease severity and the results of physical examinations and diagnostic procedures, characteristics of the abscess, family history, genetic parameters, lifestyle, diet, and tobacco and alcohol usage, all of which may have a bearing on its incidence. In addition, the diagnoses of PRPA could have been made by physicians from various subspecialties with potential for mistaken diagnoses. Further, some patients with PRPA may have been diagnosed as cellulitis or acute lymphangitis of the face and neck (L03.2), cellulitis and acute lymphangitis of the neck (L03.22), cellulitis of the neck (L03.221), or acute lymphangitis of the neck (L03.222), which may result in underestimates of incidence.

Our study suggests a higher incidence rate of PRPA in Taiwan compared to that in other countries, as well as an increasing incidence of PRPA in recent years, thus warranting further research into the possible causes such as an increased amount of performed tonsillectomy/uvulopalatopharyngoplasty procedures or lifestyle changes.

## Figures and Tables

**Table 1:** Demographic characteristics of 5779 patients diagnosed with parapharyngeal and retropharyngeal abscess (PRPA) in Taiwan during 2007–2016.

Variables	Total No/Mean	%/SD
Age, mean (SD)	52.16	16.61
Age Group (Years)		
20–44	1950	33.7
45–64	2438	42.2
>64	1391	24.1
Gender		
Male	3711	64.2
Female	2068	35.8
Geographical Location		
Northern	2818	48.8
Central	1332	23
Southern	1508	26.1
Eastern	121	2.1
In-hospital mortality	115	2.0

## Data Availability

The National Health Insurance Research Database, which has been transferred to the Health and Welfare Data Science Center (HWDC). Interested researchers can obtain the data through formal application to the HWDC, Department of Statistics, Ministry of Health and Welfare, Taiwan (http://dep.mohw.gov.tw/DOS/np-2497-113.html).

## References

[1] Woods C.R., Cash E.D., Smith A.M., Smith M.J., Myers J.A., Espinosa C.M., Chandran S.K. (2016). Retropharyngeal and Parapharyngeal Abscesses Among Children and Adolescents in the United States: Epidemiology and Management Trends, 2003–2012. J. Pediatric Infect. Dis. Soc..

[2] Buckley J., Harris A.S., Addams-Williams J. (2019). Ten years of deep neck space abscesses. J. Laryngol. Otol..

[3] Watanabe K., Kimura Y., Obara T. (2014). Gas in the retropharyngeal space: Descending necrotising mediastinitis. Lancet.

[4] Aynehchi B.B., Har-El G., Johnson J. (2014). Deep Neck Infection. Bailey’s Head and Neck Surgery-Otolaryngology.

[5] Hurley R.H., Douglas C.M., Montgomery J., Clark L.J. (2018). The hidden cost of deep neck space infections. Ann. R. Coll. Surg. Engl..

[6] Huang T.T., Liu T.C., Chen P.R., Tseng F.Y., Yeh T.H., Chen Y.S. (2004). Deep neck infection: Analysis of 185 cases. Head Neck.

[7] Klug T.E., Fischer A.S., Antonsen C., Rusan M., Eskildsen H., Ovesen T. (2014). Parapharyngeal abscess is frequently associated with concomitant peritonsillar abscess. Eur. Arch. Otorhinolaryngol..

[8] Monobe H., Suzuki S., Nakashima M., Tojima H., Kaga K. (2007). Peritonsillar abscess with parapharyngeal and retropharyngeal involvement: Incidence and intraoral approach. Acta Otolaryngol. Suppl..

[9] Brito T.P., Hazboun I.M., Fernandes F.L., Bento L.R., Zappelini C.E.M., Chone C.T., Crespo A.N. (2017). Deep neck abscesses: Study of 101 cases. Braz. J. Otorhinolaryngol..

[10] Von Elm E., Altman D.G., Egger M., Pocock S.J., Gotzsche P.C., Vandenbroucke J.P., Initiative S. (2014). The Strengthening the Reporting of Observational Studies in Epidemiology (STROBE) Statement: Guidelines for reporting observational studies. Int. J. Surg..

[11] Qureshi H.A., Ference E.H., Tan B.K., Chandra R.K., Kern R.C., Smith S.S. (2015). National trends in retropharyngeal abscess among adult inpatients with peritonsillar abscess. Otolaryngol. Head Neck Surg..

[12] Mejzlik J., Celakovsky P., Tucek L., Kotulek M., Vrbacky A., Matousek P., Stanikova L., Hoskova T., Pazs A., Mittu P. (2017). Univariate and multivariate models for the prediction of life-threatening complications in 586 cases of deep neck space infections: Retrospective multi-institutional study. J. Laryngol. Otol..

[13] Wang L.F., Kuo W.R., Tsai S.M., Huang K.J. (2003). Characterizations of life-threatening deep cervical space infections: A review of one hundred ninety-six cases. Am. J. Otolaryngol..

[14] Ridder G.J., Technau-Ihling K., Sander A., Boedeker C.C. (2005). Spectrum and management of deep neck space infections: An 8-year experience of 234 cases. Otolaryngol. Head Neck Surg..

[15] Velhonoja J., Laaveri M., Soukka T., Irjala H., Kinnunen I. (2020). Deep neck space infections: An upward trend and changing characteristics. Eur. Arch. Otorhinolaryngol..

[16] Yang W., Hu L., Wang Z., Nie G., Li X., Lin D., Luo J., Qin H., Wu J., Wen W. (2015). Deep Neck Infection: A Review of 130 Cases in Southern China. Medicine.

[17] Martinez Pascual P., Pinacho Martinez P., Friedlander E., Martin Oviedo C., Scola Yurrita B. (2018). Peritonsillar and deep neck infections: A review of 330 cases. Braz. J. Otorhinolaryngol..

[18] Panduranga Kamath M., Shetty A.B., Hegde M.C., Sreedharan S., Bhojwani K., Padmanabhan K., Agarwal S., Mathew M., Rajeev Kumar M. (2003). Presentation and management of deep neck space abscess. Indian J. Otolaryngol. Head Neck Surg..

[19] Windfuhr J.P., Chen Y.S. (2019). Hospital admissions for acute throat and deep neck infections versus tonsillectomy rates in Germany. Eur. Arch. Otorhinolaryngol..

[20] Lau A.S., Upile N.S., Wilkie M.D., Leong S.C., Swift A.C. (2014). The rising rate of admissions for tonsillitis and neck space abscesses in England, 1991–2011. Ann. R. Coll. Surg. Engl..

[21] Wang Y.P., Wang M.C., Lin H.C., Lee K.S., Chou P. (2015). Tonsillectomy and the risk for deep neck infection-a nationwide cohort study. PLoS ONE.

[22] Kim S.Y., Min C., Lee W.H., Choi H.G. (2018). Tonsillectomy increases the risk of retropharyngeal and parapharyngeal abscesses in adults, but not in children: A national cohort study. PLoS ONE.

[23] Celakovsky P., Kalfert D., Tucek L., Mejzlik J., Kotulek M., Vrbacky A., Matousek P., Stanikova L., Hoskova T., Pasz A. (2014). Deep neck infections: Risk factors for mediastinal extension. Eur. Arch. Otorhinolaryngol..

